# Dissertation on the Diseases of the Maxillary Sinus

**Published:** 1842-12

**Authors:** Chapin A. Harris

**Affiliations:** Professor of Practical Dentistry in the Baltimore College of Dental Surgery, &c.


### ARTICLE II.

*Dissertation on the Diseases of the Maxillary Sinus.* Read before the American Society of Dental Surgeons, at their third annual meeting; held in the city of Boston, July 20th, 1482. By CHAPIN A. HARRIS, M. D., D. D. S., Professor of Practical Dentistry in the Baltimore College of Dental Surgery, &c.

(Continued from page 64.)

THE condition of the teeth in the case just narrated, may not be thought to have exerted any agency in the production of the affection of the antrum, but there are circumstances connected with its history, that would seem to justify a different conclusion. The presence of the decayed teeth beneath the sinus, may not only have contributed to aggravate the morbid action lighted up in it by the cold which he had taken, but they may also have caused it to locate itself in this cavity; and the fact that the inflammation of its lining membrane and the obliteration of the nasal opening continued until they were removed, would at least, seem to warrant such an inference. That the injections were beneficial, I do not doubt, but that the cure was effected by them, no one, I think, will dare to affirm. I am far from believing that the presence of the decayed teeth was the sole cause of the disease in the antrum, but that they contributed to, and protracted it, I cannot hesitate to believe, and but for the increased excitability and perhaps actual inflammation, induced in the mucous membrane of this cavity, by the exposure of the patient to inclement and sudden transitions of weather, it is probable that the sinus would never have become affected, and I think it not unlikely, that notwithstanding the disturbance that may have been originated in it by this cause, no very serious or lasting morbid effect would have been produced, if the teeth and alveoli had been in a perfectly healthy condition.

A very interesting case of muco-purulent secretion of the mucous membrane of this cavity, occasioned by an exostosis of a superior molaris, which gave rise to an obstinate ozena, came under the observation of the author in 1839. A detailed account of it, might very properly be introduced into the present treatise, but inasmuch as the history of the case has been recently published in the "Maryland Medical and Surgical Journal," the

"Medical Examiner," and the "American Journal of Dental Science," I have thought it best to omit it.

The particulars of the following highly interesting case were communicated to me by Dr. L. Roper, an eminent dentist of Philadelphia, in a conversation which I had with him about six months since.

CASE XII.—Miss M——, a young lady from the West Indies, of about fourteen years of age, had a fistulous opening beneath the right orbit, that communicated with the maxillary sinus. By means of a probe introduced through the opening into this cavity, the apices of the roots of the first superior molaris could be distinctly felt.

Medical aid was sought at an early stage of the disease, but as no permanent benefit resulted from the treatment adopted, the young lady, at the expiration of nine months, was brought by her father to Philadelphia, and in the spring of 1831, placed under the care of the late Dr. Physick; who, suspecting that the affection of the antrum had resulted from, and was still kept up by irritation produced by the first superior molaris of the affected side, which was considerably decayed, directed her to be taken to Dr. Roper, who, concurring with him in opinion, at once extracted the carious tooth. The operation was followed by the immediate discharge of a large quantity of thick, muddy, and greenish matter. The fistula under the orbit soon closed, and without further treatment a perfect cure was accomplished in the course of a few weeks.

The foregoing are all the particulars which I could obtain concerning this interesting case; but I have no doubt that, if all the circumstances connected with its early history were known, it would be found to have resulted from inflammation of the lining membrane of the antrum, caused by irritation in the socket of the tooth that was extracted; and this opinion is sustained by the facts, that this tooth was affected with caries, and that its removal was followed by an immediate cure of the disease.

In Bordenave's collection of cases of disease of the maxillary sinus, published in the Memoirs of the Royal Academy of Surgery,* there are several cases similar to the one just narrated. I subjoin a description of the two following.

* Vide Observations vii. viii. xii. and xiii. vol. 12; 12mo. ed. pages 26, 33 and 34.

Case XIII.—A servant of the Count of Maurepes had been afflicted for six months with a fistula upon the left cheek, a little below the orbit, which penetrated the maxillary, caused by the spontaneous opening of an abscess. The third and fourth molares, (which are the first and second according as the teeth are now designated,) both of which were considerably decayed, were extracted by M. Hevin, but as there were no openings through the alveoli, he perforated one with a trocar; this opening gave vent to a great quantity of putrid sanies, and it did not close for more than a year after it was made, but the fistula of the cheek healed in about ten days.

Case XIV.—In 1717, a soldier of the regiment of Bassigny, who had for a long time a fistula in his cheek penetrating into the maxillary sinus, was treated for it at the Hotel Dieu, of Montpelier. The matter settling near the orifice of the fistula, prevented it from closing. M. Lamourier, on examining the mouth of the soldier, perceived that the second superior molaris was decayed; this he extracted and profited by the alveolar cavity, in opening the base of the sinus. The fistula of the cheek was by this means cured in a few days, but the counter opening was not immediately permitted to close.

In cases of fistula resulting simply from engorgement of the sinus, the treatment, as has been shown by the result of that in the foregoing cases, consists in the formation of a counter opening, which should always be effected at the most dependent part of the cavity, and in the removal of all sources of local irritation. Injections should also be employed.

In the cases that have thus far been presented, I have selected such as were not complicated with abscesses, ulceration of the lining membrane, or caries of the surrounding osseous walls; but to the existence of the two last, the affections on which I have just been treating, often give rise. But I will not extend my remarks further upon mucous engorgement and a purulent condition of the secretions of this cavity. The next form of disease on which I propose to speak, is abscess—an affection, differing in all its characteristics from those that have thus far been treated on.

### OF ABSCESS.

Abscess in the maxillary sinus, although very rare, does, notwithstanding, sometimes happen. The structure of the parts composing this cavity, would seem, as has been remarked by Mr. Bell, to render the occurrence improbable, and if the fact were not well established, it might perhaps be doubted. If the apices of the roots of some of the superior molares did not occasionally perforate the floor of this cavity, the occurrence of abscess in it would indeed be rare, but, as the antrum is sometimes thus penetrated, its formation here is not, after all, a matter of so much surprise. An abscess is just as liable to form at the apex of the root of a tooth penetrating this cavity, as at that of one in its alveolus, but it is very seldom that one is found seated in any other place in it. The case described by Mr. Bell is supposed to be the only well authenticated one on record. Bordenave, however, gives the history of a case of disease of the maxillary sinus,* which at the time of my previous allusion to the abscess of this cavity described by Mr. B., had escaped my recollection, so similar to this, that there can be little doubt in regard to the nature of the disease. In both instances, the affection was seated in the upper part of the antrum beneath the orbit. But, it is unnecessary to say more at present concerning these cases, as I intend in the proper place, to give a description of them.

Mr. Hullihen, in a well written article in the "American Journal of Dental Science,"† contends that abscess of the antrum as well as alveolar, consists in the effusion of pus, formed in the pulp cavity of a tooth, "between the bone and lining membrane." That this view of the subject is incorrect, is proven by the fact that abscesses are as frequently formed in the sockets of dead teeth as living ones. The matter from alveolar abscess in those cases where the plate of bone intervening between the extremity of the root or roots of a superior molaris or bicuspis, as the case may be, is thinner than the osseous walls surrounding it or them, often escapes through it into this cavity, after having first, as Mr. H. justly remarks, effused itself between the bone and lining

* Vide Mem. de l'Acad. Royale de Chirurgie, vol. 12, ed. 12mo. obs. xi. p. 31.

† Vide vol. 2, page 179.

membrane. But in this case, it cannot properly be termed an abscess of the antrum. Although the matter escapes into this cavity, and it, in consequence becomes involved in disease, yet the disease having originated in the alveolus of a tooth, which is still its principal seat, it is, in the strictest sense of the term, an alveolar abscess. It not unfrequently happens that the pus from an abscess formed in the socket of a superior molaris, discharges itself into this cavity and escapes through the nasal opening for months and sometimes for years, for, after an abscess has once formed at the apex of the root of a tooth, purulent matter will continue to be formed, though not always in the same quantity until the irritant that caused it is removed. The pulp, or ganglion as some French writers term it, may suppurate, and the matter be confined in the cavity of the tooth for a long time without causing alveolar abscess, and the purulent matter contained in the sac at the extremity of the root of a tooth, is not formed, as Mr. H. supposes, in the cavity of the organ. The alveolo-dental membranes at the apex of the root of a tooth around the nerve cord, are more vascular and are endowed with greater nervous sensibility, than at any other part, consequently the inflammatory action here is always the greatest, and it is here that suppuration first takes place.

The apices of the roots of the first and second superior molares, when they do not actually perforate the floor of the antrum, are often above its level, and covered by only a very thin shell or cap of bone, and hence in case of an abscess in one of these, although strictly alveolar, the matter is more liable to make for itself a passage into this cavity, than through the gum into the mouth. When this happens, it gives rise to inflammation of the lining membrane and causes its secretions to become more or less vitiated, and often leads to an erroneous opinion concerning the real nature of the disease.

But it is only when the root of a tooth actually penetrates the floor of the antrum, or the tubercle at its apex becomes situated in it, that the abscess can properly be said to be of this cavity. And when the root of the tooth does penetrate it, the tubercle, although formed at its apex around the nerve cord, as it is commonly called, is between the lining membrane and periosteal tissue, both of which, in the immediate vicinity become directly and at once involved in the inflammation here lighted up, and this sometimes extends itself to every part of the cavity, causing in some instances, an obliteration of the nasal opening. This however, does not often occur, but when it does, it is followed by engorgement of the sinus, and occasionally, by ulceration of its lining membrane, and disease in the surrounding bone.

It is sometimes the case, that the plate of bone intervening between the extremity of the root of a tooth, around which a tubercle has formed, and the antrum, is destroyed, and the tubercle, instead of being wholly confined within the alveolus, is forced up, as it enlarges, almost entirely into this cavity. The inflammation after having attained a certain height, is succeeded by suppuration, and the secretion of pus goes on until the sac bursts, when the matter is discharged, and, mixing with the mucous secretions of this cavity, ultimately escapes with them through the nasal opening, if it be not closed, into the nose.

As it regards the morbid effects produced upon the lining membrane and surrounding bony parietes of the antrum, by an abscess of this kind, it is of little consequence whether it be formed in it, if the matter be discharged there, or in the alveolus of the tooth that gave rise to it. The effects are about the same in one case as in the other. If the general health of the patient be good, and the natural opening of the sinus remains pervious, they seldom assume an alarming character, but under other and less favourable circumstances, the most dangerous and aggravated forms of disease to which this cavity is liable, may result from an abscess seated in either place.

*Symptoms.*—In the incipient or forming stages of abscess of the maxillary sinus, the symptoms are similar to those that characterize inflammation of the lining membrane of this cavity, or violent inflammatory tooth-ache. The pain is generally most severe in the upper part of the alveolar ridge, above some one of the molar or bicuspid teeth. From thence, it often extends to the lower part of the orbit, ear, temple, muscles of the cheek and scalp. It is more or less constant, and a throbbing is felt high up in the alveolar border beneath the cheek. If the abscess originated at the apex of the root of a tooth, this organ will appear slightly elongated and sore to the touch; the cheek in most instances is a little tumefied and more or less flushed.

The pain after having continued for several days, is succeeded by suppuration, when it immediately subsides. Slight paroxysms of cold and heat are now felt, and if the natural opening of the antrum be not closed, purulent matter will occasionally be discharged.

If the abscess be seated in any other part than the base of the antrum, the symptoms may differ in some respects from the foregoing. But if purulent matter, or mucus mixed with pus be discharged from the nostril of the affected side, when the patient inclines his head to the opposite one, or makes a sudden and forcible expiration through it, while the other is closed, the existence of abscess in this cavity, will be very conclusively indicated.

The abscess having burst, pus will be discharged from it, from time to time, for several days, which will escape through the nasal opening, except this passage has become closed or choked up with hardened floculi or other foreign matter, and then it will cease altogether, or very nearly so. The disease however, if the irritant that gave rise to it still remains, is by no means cured. A recurrence of it generally takes place, every time the patient takes cold, when all the symptoms just described will be again experienced, and each succeeding attack leaves the parts implicated in the morbid diathesis thus lighted up, in a more unhealthy condition, and as a consequence more susceptible of being acted upon by morbid irritants. Suppuration also, at each successive attack takes place, and the pus gradually assumes a worse character.

*Causes.*—It will not be necessary to say much concerning the causes of abscess of the antrum. It will be sufficient to state, that they are the same as those of tooth-ache, inflammation of the alveolo-dental periostea and inflammation of the lining membrane of this cavity, and it is to the presence of one or other, or both of these that it is attributable. And these may be excited, by caries of the teeth, a blow upon them, or a dead or loose tooth, or a blow upon the cheek and by exposure to sudden changes of weather, as from heat to cold, &c. Other causes may sometimes be concerned, but the foregoing are the principal, and all that it is necessary to enumerate here.

*Treatment.*—In the cure of abscess of the maxillary sinus, as well as that of a muco-purulent condition of its secretions or engorgement, the first and most important indication to be fulfilled, is to obtain a vent for the matter, from the inferior part of the cavity. The best method of doing this has before been described, and it will be unnecessary to recapitulate the directions that have been given for the performance of this operation.

The formation of abscess in this cavity might however, in almost every instance be prevented, by the timely adoption of proper treatment. On the occurrence of severe, deep-seated and throbbing pain in the upper part, or just above the alveolar ridge in the region of the antrum, such as has been described as attending the formation of abscess in this cavity or in that of the alveolus of a superior molaris, if the tooth directly beneath the place where it was first felt, be considerably decayed, or its lining membrane exposed, or if it be dead, loose, or its socket much diseased, it should be immediately extracted. By this simple operation, the formation of abscess not only in the socket of the tooth, but also in the antrum, may, in almost every instance be prevented. If however, it be not followed by an immediate subsidence of pain, leeches should be applied to the gums and fomentations to the cheek. If the patient be of a full habit, and if there be any general febrile symptoms, saline purgatives may also be employed with advantage. But in the majority of cases, the extraction of the tooth will be all that is required to arrest the progress of the disease.

The curative indications, if the abscess be of recent formation, and has resulted from the presence of a diseased tooth, are similar to the preventive. The first thing to be done is to remove the tooth that caused it, and if this operation be not delayed too long, it, in most instances, will be all that is necessary to effect a cure. In addition to this, Mr. Hullihen recommends the perforation of the antrum;* but in those cases where the abscess has formed at the apex of the root of a molaris, this is not necessary, because in all such cases, the alveolus communicates with this cavity, so that on the removal of the tooth, there will be a sufficiently large opening into it; and besides, the tubercle or sac

* American Journal of Dental Science, vol. ii. p. 182.

although situated within the sinus, is in nearly every instance brought away with the tooth.

But, when the abscess has been of long standing, and the lining membrane of the antrum become seriously affected, in addition to the removal of the tooth, other treatment will have to be resorted to. The opening into the antrum, if necessary, should be enlarged, and it should be prevented from closing until the health of the lining membrane is restored; and for the promotion of this, injections, such as have been previously recommended,* will be found serviceable.

In cases of simple abscess of the antrum, seated at the apex of the root of a superior molaris, I have never found it necessary to adopt other treatment than the foregoing. It may, however, in some instances, be necessary to remove more than one tooth, even though that be the one that gave rise to the abscess. The following case presents an example of this kind.

Case XV.—Miss E. M—, æt. seventeen, of a scrofulous habit, was placed under my care in the spring of 1837, for the purpose of obtaining relief from a severe deep-seated pain in her right cheek, apparently a little above the first superior molaris, and which was supposed to result from a diseased condition of several teeth on that side of the upper maxillary. The pain, although very severe, seldom lasted more than three or four days. She had experienced several attacks of it,—the first about eighteen months before I saw her, and its subsidence, every time, was followed by occasional discharges of purulent matter from the nostril of the affected side. These discharges, at first, continued only for three or four days, but they lasted longer after each successive attack, and became more acrid and offensive.

Three days previously to my seeing her she had had an attack, and the inflammation at the time had nearly reached a crisis. Her cheek was slightly swollen and considerably flushed, and an exceedingly painful throbbing sensation was felt in the region of the malar apophysis. I directed four leeches to be applied to th gum covering the lower part of this, beneath the cheek, fomentations to the face, and pediluvium before going to bed. These, I was informed, had been prescribed in preceding attacks, and that

* Vide page 56.

only very temporary benefit had been obtained from them, I, in consequence, contented myself, knowing that the inflammation would soon run its course, by advising the application of anodyne fomentations to the face.

The next day I saw her, and the pain had nearly subsided, but there was considerable inflammation and sponginess in the gums around the superior bicuspid and molar teeth. The first molaris was very sensitive to the touch. The morning of the second day after I saw her, matter resembling pus, and of a fetid odor was discharged from the nose. Believing that this came from an abscess in tne antrum, caused by the presence of the first superior molaris, I advised its immediate removal, to which, after considerable persuasion, she submitted. As I had suspected, this proved to be the tooth which had occasioned the mischief. At the extremity of its two outer fangs, which were almost in contact, was a tubercle the size of a large pea, and there was an opening into the antrum through which a small goosequill could be passed. A considerable quantity of bloody mucous matter streaked with pus, immediately escaped from it. I urged upon the patient, the importance of having several other decayed teeth removed, though not as necessary to the cure of the affection to which she had been subject, but she would not submit to the operation.

Three weeks after the extraction of the tooth I again saw her. She had not since experienced any pain; the alveolus had closed, but a glairy fetid matter was occasionally discharged from her right nostril. The alveolus was re-opened by cutting away the fleshy granulations that had filled it, and a small quantity of matter such as I have just described, came away. Injections were now employed of diluted tincture of myrrh, and rose water with a small quantity of sulphate of zinc. The opening was prevented from closing, and the use of the injections persisted in for three or four weeks, when the bougie that had been inserted in the alveolus to prevent it from filling up, was left out. The opening soon closed, and in the course of ten or twelve days, a glairy fetid matter was again occasionally discharged from the nostril of the affected side, and again a vent was procured for it through the socket of the tooth that had been extracted. This was now kept open for five or six weeks, when it for the third time was permitted to close, and for two weeks there were no signs of a return of the discharge of fetid glairy mucus from the nostril; but at the expiration of fifteen or sixteen days it became apparent that the mucous membrane of the antrum was not restored; its secretions again became fetid. Suspecting that the diseased condition of the teeth, gums, and alveolar processes beneath the cavity exerted a morbid influence upon it, I, a second time, urged the removal of the first bicuspis, and the second and third molaris, which were all so much decayed as to render their restoration out of the question. With much persuasion the consent of my patient was obtained, and the teeth were at once extracted.

Four weeks after, the secretions of the antrum had become healthy, and they have since remained so.

The morbid condition of the mucous membrane of the antrum, although in this case it had no doubt resulted from the abscess that had formed at the apices of two of the roots of the tooth first extracted, was subsequently kept up, by the irritation of the alveolo-dental membranes, occasioned by the other decayed teeth.

Before I conclude my remarks upon abscess of this cavity, I will give the history of the two cases to which allusion has before been made, the one is narrated by Mr. Thomas Bell,* and the other by Bordenave.† The following detailed statement of the first I quote from his treatise on the teeth.

CASE XVI.—"Mary B——, aged eighteen, of an unhealthy and somewhat strumous aspect, of languid disposition, and of retiring and timid habits, came under my care on the 3d of January, 1817, in consequence of severe and continued pain on the left side of the face, of a dull heavy character, and, apparently deep-seated; but occasionally darting in acute paroxysms, across the face towards the nose. The cheek was swollen, and the palate somewhat enlarged. About a year before the first superior molaris of that side had been extracted on account of severe pain in the face, but without producing any relief, and the pain was consequently attributed to rheumatism, from which com-

* Anat. Phys. and Diseases of the Teeth, p. 270.

† Mem. de l'Academie Royale de Chirurgie, ed. 12mo. vol. xii. p. 31, Obs. 11.

plaint she had long suffered to a great degree, in the shoulder, hip, and other joints, and for which she had been under the care of many medical practitioners, both in London and Bath, having been sent to the latter place for the use of the waters. When I first saw her, the general health was much deranged: the stomach, bowels, and liver performed their functions very imperfectly; and the uterus partook of the general sluggishness of the system, menstruation being almost wholly suppressed, and the periods only indicated by increased indisposition, and especially by an exacerbation of the pain in the face.

No discharge had taken place from the nose, but, from the nature and situation of the pain, the direction of its paroxysms, the enlargement of the cheek and palate, and from an occasional trifling discharge of pus from the alveolus of the tooth which had been extracted, I could not doubt that the antrum was the seat of the disease. On examining the teeth, I found that the second bicuspis also was diseased, and as it had at times occasioned considerable pain, I extracted it with the view of removing every possible source of irritation.

Six leeches were ordered to be applied to the face, and afterward the continued application of a cold lotion. Medicines were also administered with reference to the general health, both as regarded the digestive and uterine functions; and on January 7, I determined on puncturing the antrum. I consequently introduced the trocar through the anterior alveolar cavity of the first molaris, and found that when the instrument came in contact with the lining membrane, the most acute pain was produced, indicating the existence of a high degree of inflammation in that structure. On withdrawing the trocar, when the antrum was freely opened, I was surprised, and a little disappointed, at finding that not the smallest discharge made its appearance. There was a small quantity of glairy mucus, but nothing more. I introduced the blunt end of a probe, and found that the opening was quite free; but on passing it upwards towards the orbit, its passage was resisted by a firm elastic substance, which gave the impression that a solid tumour existed in the upper part of this cavity, and which produced intolerable pain, on being pressed with the probe. I now injected some tepid water, and found that the nasal opening was pervious, as the water passed freely into the nose. As the operation had produced a considerable increase of pain, and as the parts appeared a good deal inflamed, I ordered six leeches to be applied, the bowels to be freely opened, and an opiate to be taken at night.

January 9.—The pain has been extremely severe ever since the operation, with scarcely any mitigation excepting for a few hours after the application of the leeches. A probe now introduced into the antrum, met with similar resistance, but much nearer the orifice than before, proving that the tumour had increased; and on injecting warm water, it no longer passed into the nose. The leeches, the aperient, and the opiate were repeated.

January 11.—The pain has continued without cessation, and no sleep has been procured by the opium. The inflammation is not apparently reduced. Pulse one hundred, small and feeble. The palate is a little enlarged, but not more so than might be accounted for, by the thickening of the integuments by inflammation, I could now distinctly feel with a probe, that the tumour was not only increased in size, but that it had become softer, yielding in some measure to pressure, and conveying the impression that it contained fluid. I therefore introduced a sharp-pointed instrument, which, with a little force, pierced the tumour, and a gush of pus instantly took place, with immediate relief to the symptoms.

Here, then, was a sac containing pus, existing doubtless as a distinct cyst, the result of inflammation in the membrane; for it is scarcely probable that the membrane itself had become separated from its attachment by the formation of pus between it and the bone. That the fomer was the true situation of the disease, may be inferred from the fact that no subsequent caries of the bone took place, which would, undoubtedly, have been the case, had the matter been formed in contact with the bone; and it could scarcely have been produced between the mucous membrane and the periosteum, as these two structures, though essentially distinct from each other, are inseparably connected.

The pus continued to be discharged for a day or two, and then entirely ceased. On passing the probe a week after the former operation, I found the same resistance as before, and in the same situation; the cyst was again punctured, and again the pus was discharged. This alternation of the repletion and evacuation of the cyst regularly recurred for a considerable time, but the opening into the nose did not again become stopped. The general health, however, in the meanwhile, improved, and the pain in the face was greatly diminished, returning only, with any degree of violence, when the cyst was full.

At length the repeated perforation of the sac, followed by the use of strong astringent injections, and aided by the remedies that were directed to the state of the general health, restored the antrum to healthy condition; the menstrual disturbance was by degrees entirely obviated, and the stomach at the same time assumed its healthy function; but it was two years from the time when I first saw her before she had recovered her health, which at the best was never robust."

The case described by Bordenave, is, in many respects, similar to the foregoing. The following account of it is taken from the Memoirs de l'Academie Royale de Chirurgie.

Case XVII.—The subject of the present case was a young Russian of about ten years of age. He was seized with acute pain in his teeth, which was soon followed by tumefaction of the cheek. An abscess developed itself two days after above the second molaris, and this was succeeded by a cessation of pain. For the cure of this new affection, the extraction of the decayed tooth, as also the two neighbouring ones, were decided on. Through the alveolus of the first, the antrum was then perforated. It was afterwards injected with appropriate remedies, and the opening prevented from closing by the introduction of pieces of cotton into it. The treatment having been conducted thus far, the patient was put under the care of Bordenave. The injections when thrown in with force passed in part through into the nose, and the liquid which returned brought with it thick and fetid matter. From time to time the patient experienced a sensation in the sinus near the orbit, like what would be produced by the bursting of something, and soon after each of these paroxysms, there was a discharge of a large quantity of fetid pus. When the escape of this matter was prevented by the contraction or closing up of the opening, a slight uneasiness was experienced. To prevent this, Bordenave dilated the opening with prepared sponge, and placed into it a silver canula pierced with several holes, for the purpose of permitting the matter freely to escape. Time after time the matter became thick and lumpy, and on an occasion when the canula was withdrawn to be cleaned, the patient, by sucking, effected a discharge of foreign matter. At the expiration of six months, the matter having become of a better quality, the use of the canula was discontinued. Its replacement however, as Bordenave supposed, having again soon become necessary, it was restored and its use continued for nearly two years, at the expiration of which time the quality of the matter had become healthy. The patient ceased to feel any uneasiness in the sinus, but as the opening did not close, it was slightly cauterized. It now soon healed, and at the end of 1758, the disease had terminated. For the correctness of the foregoing statement, Bordenave refers to M. Morand, who visited the patient with him.

It is highly probable that, had this case been treated in the manner in which Mr. Bell treated his, a cure would have been much sooner effected. I should think it highly important that the matter should have free egress both from the cyst in which it is secreted, and from the antrum as soon as it escapes from that into it.

Finally, that abscess does occasionally form in other parts of this cavity than the base, is conclusively proven by the two last cases. It is true, these are the only ones of which we have any account, yet, nevertheless, they establish the fact that it is possible for them to occur in any part of the sinus.

### OF ULCERATION OF THE LINING MEMBRANE.

This is not an idiopathic affection. It is always, I believe, symptomatic of some other morbid condition of the mucous membrane of this cavity, and often gives rise to some of the worst and most aggravated forms of disease that are ever here met with. It is not therefore a simple disease, but is complicated with the one that caused it, and often with some other to which it has given rise. I shall treat of it, however, as a separate affection. Its attacks are preceded by a purulent condition of the fluids of the antrum, and are often followed by fungi and sometimes by caries of the surrounding osseous walls. The membrane covering the floor of the cavity, is usually first attacked, but ulcers having formed here, they generally soon extend themselves to other parts of the sinus.

Ulceration of the lining membrane of the maxillary sinus is frequently complicated with ulceration of the lining membrane of one or both of the nasal cavities; and, ulceration in the antrum is sometimes mistaken for ulceration in the nose, but it is not easy thus to mistake the seat of the disease. The existence of ulcers in the former can only be inferred from certain signs, but when seated in either of the latter, they can almost always be seen, and besides, the matter secreted by them exhales a less fetid odor than does that of ulcers of the antrum maxillare. The reason of this is obvious. The air that finds its way to this cavity is retained in it a long time and consequently becomes more highly impregnated with the fetor of the matter secreted in ulcers situated here, than it does with that of ulcers of the nose, over which, it is almost constantly passing. This of itself, as has been justly remarked by Deschamps,* will enable us to determine, almost to a certainty, the seat of the disease. But there are other signs that will assist us in ascertaining its location. The foregoing, however, are sufficient, especially when taken in connection with the symptoms that precede the formation of the ulcers.

Ulcers of the maxillary sinus present as great a variety of character as do those of other parts of the body. Their nature is determined by the state of the constitutional health and the causes that produce them. But it is not necessary to go into a minute description of the various kinds of ulcers that are here met with. It will suffice to state that they for the most part partake of the disposition of the subject in which they occur, and that the following varieties have been met with; namely, the simple, or that resulting from mechanical injury; the fungus, the scorbutic, venereal, cancerous, gangrenous, scrofulous, inveterate, carious, &c. &c. Sir Everard Home divides ulcers into six kinds, each of which being determined by the nature or condition of the part that it is situated in.† The first, are ulcers in parts endowed with sufficient strength or curative power to effect their restora-

* Maladies des Fosses Nazales, Sec. 2, Art. vi. p. 262.

† Cooper's Surgical Dictionary, vol. ii. p. 381.

tion. The second are those situated in parts too weak to effect a recovery. The third are ulcers in parts having too great an action for the formation of healthy granulations. The fourth are those seated in parts possessed of too indolent an action, whether arising from the state of the parts or general constitutional health. The fifth are ulcers located in parts that have acquired some specific diseased action. The sixth and last, are those situated in parts that are prevented from giving out healthy granulations, by a "varicose state of the superficial veins of the upper part of the limb." But, these remarks are applicable only to ulcers in general; yet, as those of the maxillary sinus often present characteristics similar to those of other parts of the body, they may not be deemed inappropriate here. The kind of ulcer last noticed, however, never occurs in the antrum.

In the simpler species of ulcer, the matter is of a thick consistence and nearly white. but as the disease increases in malignancy, it is thinner and varies in appearance from transparent to a dirty brown, yellow or black.

*Symptoms.*—Many of the signs attendant upon ulceration of the mucous membrane of the maxillary sinus, are similar to some that accompany other affections of this cavity, as for example, deep-seated heavy pain in the cheek, the occasional escape of matter into the nose, &c. &c. But in addition to constant pain in the region of the antrum, the following may be mentioned as signs indicative of ulcers of this cavity. The escape of a fetid sanies into the nose on the patient's inclining his head to the opposite side, or through an opening which it has itself effected, or that has been formed by art for its escape. Also, the traversing of the ulcer from the interior through the bony walls of the cavity and external soft parts. An opening of this sort may be effected through the cheek, near, or even into the orbit, which last has often happened; at other times it is effected through the canine fossæ or palatine arch. Moreover, the matter escaping from the sinus, often has floculi mixed with it, which is never the case, in simple muco-purulent secretion of the sinus. These floculi sometimes, as has been before stated, choke up the natural opening of the cavity and cause its secretions, together with those of the ulcers to accumulate, and distend its osseous walls until they ultimately give way, or an opening is formed for their escape. It occasionally happens however, that the floculi that have gotten into the nasal opening, and thus prevented the egress of the fluids secreted here, after choking up this conduit for a long while, suddenly give way and permit the matter to pass out into the nose.

When the ulcer is of a fungus character, the matter secreted by it, is thin and of a dark brown or blackish colour; and it has mixed with it blood and pus.* It is also, says Deschamps, slightly painful, and can only be distinguished from other ulcers by the introduction of the bougie into the sinus, and that like polypus, it is capable of spreading itself and penetrating every opening that will give it passage; but in consequence of its being of a softer consistence, it makes less impression upon the surrounding parts.

If the ulcer be of a cancerous nature, the pain will be sharp and lancinating and affect the whole of the side of the face; the matter will be serous, very fetid, and streaked with blood. If it is discharged through the natural opening into the nose, it will cause the pituitary membrane of the nasal cavity of the affected side to become exceedingly irritable, sensitive to the touch, and ulcerated. The bones of the affected side of the face soon become softened or carious, the teeth loosen, and the external soft parts inflame and ultimately ulcerate; openings are formed into the sinus, fever of a low grade supervenes, and ultimately death closes the scene.

*Causes.*—A degenerated or altered state of the secretions of this cavity, is said to be the most common cause of ulcers in it.† This may doubtless be an exciting cause, and it may be one of the most frequent exciting causes, but were it not favoured by a constitutional predisposition or tendency, it would not perhaps often give rise to them. Local irritation, whether produced by an altered condition of the fluids of this cavity or by the presence of decayed or dead teeth, the roots of teeth, or a blow upon the cheek, may be, and doubtless is, the exciting cause of ulcers in the mucous membrane of the maxillary sinus. This, however, in a subject of good constitutional health, would have to be very severe and continue for a long time, to result in ulceration of this membrane, and even then, a cure would soon be effected by the

* Maladies des Fosses Nazales, sec. 2, Art. vi. p. 263.
† do. do. do. p. 159.

restorative efforts alone of the economy. It is only in bad habits, or debilitated constitutions, that malignant ulcers are often met with in the maxillary sinus.

Deschamps, although he acknowledges that diseased teeth often exercise a morbid influence upon this cavity, and that the apices of the roots of these organs are sometimes in contact with its mucous or lining membrane, seems nevertheless to doubt that they have any agency in the production of ulcers here. His reasoning upon the subject, however, is far from satisfactory. While he admits that by the contact and adhesion of the dental periosteum and mucous membrane of this cavity, by the penetration of its floor by the roots of teeth, inflammation and ulceration may be produced, he denies that this can be positively proven. Now, although we may not be able to adduce positive evidence in confirmation of it, the circumstantial proofs which we have, are so clear and strong, that no candid inquirer can for a single moment doubt that the disease in question, when favoured by a bad habit of body, often results from dental or alveolar irritation. And, in reply to the question which he a little further on propounds, "How can the extraction of a tooth be of service in the subduction of inflammation of the mucous membrane with which the dental periosteum is only simply in contact,"* I answer that by this operation a constant source of irritation may be, and often is removed. But, ulcers having absolutely formed here, a cure cannot always be effected by the removal simply of the exciting cause.

But, inflammation of the lining membrane of the maxillary sinus, and as a consequence, an altered condition of its secretions, may, it cannot be denied, be produced by other causes than irritation resulting from a diseased condition of the teeth, and it is to this, that ulceration in it, is attributable.

*Treatment.*—As in the case of mucous engorgement of this cavity, the first indication of cure is to give egress to the purulent matter, and in this as in the other affection, the opening should be formed at the most dependent part of the sinus; and this should be effected in the manner as before described through the alveolar border or rather alveolus of a molaris. It should be made large enough to admit the little finger, and if there be any teeth so much affect-

* Maladies des Fosses Nazales, sec. 2. Art. vi. p. 259.

ed as to be productive of irritation to the parts subjacent to the antrum, they should at the same time be removed.

Free egress for the matter having been obtained, and all local irritants removed, the antrum should be injected from time to time, with gently stimulating and detersive fluids. This, in cases of simple ulcer, if the constitutional health be not seriously impaired, will often, as is proven by the result of the treatment detailed in the history of the following case, be all that is necessary to effect a cure.

CASE XVIII.—Mrs. R——, æt. about twenty-five years, of a scrofulous habit, having been affected for several months with a pain in her left cheek, which at times was very severe, and supposing it might be connected with her teeth, applied to me in the winter of 1836, for the purpose, if possible, of obtaining relief. She informed me that she had been several times temporarily relieved by a sudden discharge of matter from the nostril of the affected side, after sneezing, and once after a violent expiration through this cavity of the nose while the other was closed. I at once suspected the disease to be ulceration of the mucous membrane of the antrum, and a purulent condition of its secretions.

On examining her mouth, I found the most of her teeth to be more or less affected with caries. The crowns of the first and second superior molares of the left side were nearly destroyed, and over the roots of the second, externally, was a fistula from which, matter had at times been discharged, as I was informed, for several years. This communicated with an abscess partly between the apices of its three roots; and, as neither this nor the first molaris was of any service, their restoration being wholly impracticable, and as both obviously exercised a morbid influence upon the neighbouring parts, I advised their immediate removal. To this operation, she readily submitted, and it having been performed, I at once perforated the antrum through the socket of the second tooth, by means of a trocar suited to the purpose. The withdrawal of the instrument was followed by the discharge of more than a table-spoonful of thickened fetid mucus, streaked with blood and pus. The opening was enlarged to about the size of a goosequill and the sinus injected with tepid water and the tincture of myrrh. The opening was prevented from closing by means of a bougie prepared for the purpose. Whenever this was removed during the first eight or ten days a small quantity of whitish pus was discharged with the mucus that came away. The injections were continued for about four weeks, and at the expiration of this time, as the secretions of the antrum had ceased to be offensive, and as they were no longer mixed with pus, the bougie was left out and the opening permitted to close. A complete cure was effected.

If the ulcer be of a fungus nature, the employment of escharotics, and sometimes even the actual cautery becomes necessary, and this last should be repeated until the fungi are completely destroyed. With regard however to the employment of escharotics, such as the nitrate of silver, blue vitriol, &c. &c. for the purpose of destroying luxuriant granulations in ulcers, Sir E. Home* is of the opinion that it is better to combine them with some other substance, so as to prevent them from immediately destroying the granulations. He believes that when this is done, the surface of the ulcer underneath, is more liable to reproduce them, than when they are removed by absorption, and it is for this reason that he prefers, in the employment of caustics, to mix them with other substances, so that they shall only exercise a strongly stimulating effect, and thus cause the granulations to be gradually removed by the action of the absorbents.

The surface of the ulcer should, if practicable, be kept clean by means of dosils of dry lint or pledgits spread with some simple ointment. The treatment of ulcers of this cavity, however, is usually attended with more difficulty, on account of their concealed situation, than those of most other parts of the body. Among other things, Deschamps recommends injections of a decoction of quinine. In many cases a lotion of sulphate of zinc may be used with advantage. But the remedies to be employed in the treatment of ulcers of the maxillary sinus, as in the treatment of ulcers of other parts, should be varied to suit the indications of each particular case. In debilitated subjects, tonics, such as quinine and preparations of steel are said to be highly serviceable. There are some cases in which mercurials are highly serviceable. Strict attention should always be paid to the

* Cooper's Surgical Dictionary, vol. ii. p. 382.

regimen of the patient, and such general treatment adopted as may be best calculated to restore the constitutional health, for upon this, the cure of the local affection often depends.

If the ulcer be of an irritable nature, warm fomentations, conveyed to the interior of the antrum by means of a properly constructed funnel, of a decoction of poppy heads, chamomile flowers, or the leaves of hemlock, will often prove beneficial in soothing the pain. Tincture of myrrh, diluted, or a decoction of walnut leaves may often be advantageously employed as injections in cases of indolent ulcers,—the last of which, is recommended as an application to ulcers of this character in other parts of the body by Hunezawsky,* and both of which are favourably spoken of by Sir E. Home.† This last named writer recommends "diluted sulphuric acid and the juice of the powder of different species of pepper in a recent state," and also nitrous acid diluted with water. The unguentum hydrargyri nitrate, mixed with lard, and the ceratum resinæ, and the unguentum elemi, mixed with the balsam of turpentine, are also recommended, but the application of ointments to ulcers of this cavity is always attended with inconvenience, and it is on this account that they are less easily cured when seated here than when situated in other parts of the body.

Many of the ulcers of the maxillary sinus are regarded as incurable, as for example, such as are of a cancerous nature, and ulcerated fungus hæmatodes. But although the resources of surgery have hitherto, in most instances, proved inadequate to the cure of these formidable diseases, yet nevertheless they should be put in requisition, and we should endeavour to combat them by every means in our power. Deschamps says, the interior of the antrum should be exposed at the commencement of the disease. He recommends the formation of a large opening, if the alveolar ridge be healthy, above it, if not, through it.‡ As much of the cavity as possible should be exposed. This done, he directs, if there be a cancerous tumour, that it be as thoroughly extirpated by means of a curved and flat bistory or curved scissors as possible. All that may have escaped removal by this means he says,

* Acta Acad. Med. Chirurg. Vindob. t. 1. 1788.
† Cooper's Surgical Dictionary, vol. ii. p. 385.
‡ Maladies des Fosses Nazales, sec, ii. Art. vi. p. 265.

should be touched with the actual cautery. These, he says, are the only remedies "to be employed when the membrane is in a state of cancerous ulceration." The surgeon, he adds, "should destroy the parts in such a way as to leave only the osseous surfaces, and he should pay some attention to these bony parts, which also he should carefully cauterize."* The disease having been thus removed, the surrounding osseous walls that have been cauterized will soon exfoliate, when a chance for a cure will be afforded, and of which, if the neighbouring parts have not been invaded, nature will avail herself. The administration of soothing and anodyne medicines are also directed. Arsenic has been employed with advantage both as an external and as an internal remedy in ulcers of this kind.

There are other kinds of ulcers of this cavity, but it is not necessary here to describe the treatment for each of the various forms which this description of disease puts on. Particular and ample directions for that of each are laid down by writers on affections of this kind, and though they may not have special reference to their occurrence in the antrum maxillare, they will, for the most part, be found as applicable to them here, as when they are seated in other parts of the body.

The following case of fungus ulcer complicated with alteration of the walls of the sinus is taken from Bordenave's collection of observations on the diseases of this cavity, in the Memoirs of the Royal Academy of Surgery.* Although the history of the case, in its translation, is abridged a little, yet no important fact connected with it is here omitted.

CASE XIX.—The subject of this case was a women twenty-six years of age, and who having exposed herself, while in a critical state of health, to cold air, was in 1759, attacked with acute pains in the left side of her upper jaw, in the alveolar ridge of which, were the roots of several decayed teeth. The following day her jaw was much swollen, and although the pain ceased in a few days, the swelling still continued, without any change in the appearance of the skin; yet nevertheless, her face was deformed in shape. The orbitary apophosis of the maxillary bone became elevated, and the substance of the bone softened. The interior of

* Maladies des Fosses Nazales, Sec. 2. Art. vi. p. 266.

† Obs. xvii. in tom. 12th, 12mo. ed. p. 56.

the nose was also affected and the opening of the sinus into this cavity was closed. The matter collected in the antrum began to escape, twenty-two days after the attack, through the alveoli.

In January, 1761, the symptoms becoming more aggravated, she went to Paris in search of medical aid. M. Beaupreau was consulted, and on examining the affected parts, determined on the extraction of the decayed teeth, which were considerably broken. They however adhered so firmly to their alveolar cavities that he could not move them without shaking their sockets. This deterred him from proceeding with the operation as he had begun, and he resolved to remove the whole of the alveolar border with a bistory, from the lateral incisor to the first molaris, and in this way remove the teeth with the bone. This done, he made a section of the bone, which had become softened, with a pair of scissors, in the direction of the cuspidatus. The antrum was much dilated; its membrane fungus and ulcerated. He then treated it with detersive injections, adhesive dossils, covered with digestives, composed of the oil of turpentine. In addition to these, mercurial ointment and red precipitate were used. Alterative pills and beverages clarified with cress, were also prescribed; and this treatment was successful, for, five days after it had been commenced, the tumour had perceptibly diminished, the pus became of a better quality and less in quantity. At the expiration of two months the discharge became mucous. Injections of lime water, at first strong, and afterwards milder, were used. The natural opening was closed, and it continuing impervious, an opening through the base of the sinus was preserved. At the expiration of two months the parts had recovered, and the general health of the patient was restored.

The medical treatment, in the foregoing case was very proper; it accorded with the curative indications of the disease, but the surgical, evidently involved a greater sacrifice of parts than was absolutely called for. The extraction of teeth was not, however, as well understood at that time as at present, and it was to the want of proper knowledge and skill in this department of surgery, that the removal of so considerable a portion of the alveolar ridge was had recourse to. It is often necessary, it is true, to make a very large opening into the sinus, but it is seldom requisite to make one as large as the one that must have been made in this instance; and although nearly the same treatment was adopted in a case of a somewhat similar nature by Bourdet,* the practice is nevertheless objectionable. When the subjacent bone and alveolar border are in a carious or necrosed state, their removal would be proper, and there are diseases that occur in this cavity which render the operation necessary, but in neither of the cases just noticed were the bones carious, nor was the nature of the diseases such as to require so large an opening. In the first case, the outer wall of the sinus, as would seem from the description given, was softened, but in the other, Bourdet says the bones were not diseased.

It sometimes happens that when the inferior opening is very large, it never closes, and when the natural opening becomes obliterated, it is requisite to preserve one through the alveolar ridge; in either of these cases the employment of an obturator is necessary to prevent particles of food and extraneous matter from getting into the sinus. But of these, I shall hereafter speak.

The history of many more highly interesting cases of ulceration of the mucous membrane of this cavity, might be here introduced, but as this form of diseased action is so often complicated with caries, necrosis and other alterations of its osseous walls, I have thought that it would be as well to reserve them until I came to treat of those affections; which I shall now proceed to do.

### OF CARIES, NECROSIS AND SOFTENING OF ITS BONY PARIETES.

Various opinions concerning the pathological peculiarities of the several morbid conditions of the osseous tissues of the body have been advanced; but it is not my intention, at this time, to notice any of them, further than may be necessary to a correct explanation of the curative indications of the diseases of those of the maxillary sinus.

The bones, endowed with vitality, are, like other parts of our organization, liable to disease. They are furnished with blood-vessels, nerves and absorbents, from which they derive nourishment, and, the teeth excepted, the power of undergoing various

* Dissertation sur les Depots du Sinus Maxillare, obs. 111, p. 13.

changes. These attributes however are more peculiar to some bones than others. The power of recuperation, for example, is possessed in a much higher degree by cylindrical than by flat bones, and the teeth are entirely destitute of this attribute. Unlike other bones, they are incapable of repairing any loss of substance which they may sustain from mechanical violence or other causes. Nor do their morbid conditions, exostosis and necrosis excepted, bear any resemblance to those of other bones.

Excepting then, the affections of these organs, the diseases of the bones are regarded by most writers, as analogous to those of the soft parts, and like which, they too, are said to be susceptible of being affected by constitutional vices.

By the ancients, caries and necrosis were regarded as one and the same disease. Modern surgeons, however, discriminate one from the other. Caries of bones, is represented as analogous to ulceration of soft parts, while necrosis is said to be similar to mortification. Caries does not at once destroy the vitality of the bone; a diseased action, tending to soften and otherwise alter the texture of it, is often, for a long time carried on; its cells are filled with fungous flesh, while there is constantly discharged from the affected part, a dark-coloured fetid sanies. Necrosed bone, is deprived of all vitality. The whole or only a part of a bone, (except when it occurs in a tooth, and then the whole organ perishes,) may be affected by it.

Besides caries and necrosis, there are other morbid conditions to which bones are liable, but I shall not, at this time, speak of but one, and that consists in a softening of their texture, which, by surgeons, is designated by the name of mollities ossium. This softening is supposed by some, to be owing to the absorption of the phosphate of lime of these tissues, but I am inclined to the opinion that it is occasioned by the chemical decomposition of this earthy material, by some morbid or altered fluid exhaled or poured out upon the bone or part of the bone thus affected.

Having premised these few general remarks, I shall proceed to notice more particularly the affections to which I have just adverted as occurring in the walls of the maxillary sinus. The bony parietes of this cavity, and sometimes the whole of the subjacent alveolar border and even that of the superior maxillary, and the nasal, palatine and orbital bones, as well as some that belong to the base of the cranium and the malar bone, are involved in caries or necrosis. Mollities ossium, though it rarely occurs in the alveolar ridge, frequently affects the walls of the sinus. Caries may affect a considerable portion of both for a long time, without completely destroying the vitality of the diseased parts, and during its continuance a fetid sanies will be discharged from one or more fistulous openings through some part of the cheek, alveoli, gums, palatine arch, or into the sinus, and from thence through the natural opening into the nose. The disease however eventually terminates in the decomposition and death of the parts affected by it, and then by an operation of the economy this is separated from the living bone and thrown off, or in other words, is exfoliated. But although caries ultimately causes the death of the bone or part of the bone affected by it, it does not always precede the destruction of vitality in osseous tissues. The occurrence of necrosis, therefore, although it may result as a consequence of caries, is not necessarily dependent upon it.

When the parietes of the antrum or alveoli are affected by necrosis, the soft parts in contact with the diseased or dead bone, inflame, ulcerate and discharge a fetid ichorous matter. The gums sometimes become gangrenous and slough. The destruction of the vitality of the osseous parts often progress very slowly, and thus piece after piece is exfoliated until the disease is arrested.

But besides these affections, it not unfrequently happens that the osseous parietes of the antrum, are so softened as to be easily bent. This alteration of the bone, as well as the others just noticed, are, in nearly every instance preceded by some other affections of this cavity.

The annoyance occasioned by caries and necrosis of the bony walls of this cavity or of the alveoli, to the unhappy patient, is very great. The fetor of the sanies is sometimes almost insufferable, and moreover, this matter often excoriates and inflames the parts with which it comes in contact to such a degree, as to cause them to become exceedingly sensitive and not unfrequently to ulcerate.

*Symptoms.*—It is sometimes difficult to distinguish caries and necrosis of the bony parietes of the antrum from some of the affections that seat themselves within this cavity. They therefore often exist for a long time without being suspected. But the signs that indicate mollities ossium or softness of the walls of this cavity, are such, as not to be easily mistaken for those of any other affection. In this disease, the walls of the sinus yield to pressure, and regain their former shape when the pressure is removed. Its existence, therefore, may always be known by these signs, and as these are sufficient, it is not necessary to enumerate any of the others by which it is characterized. But caries and necrosis not being so easily detected, often make considerable progress before their existence is ascertained. The fetor and appearance of the matter discharged, do not always furnish a diagnosis that can be relied upon, inasmuch as some of the diseases that occur within this cavity, cause its secretions to become equally as offensive, as the sanies resulting from caries or necrosis, and not unlike it in appearance. Their existence however may in most instances be inferred, from the discharge of a dark-coloured fetid sanies, but the exfoliation of pieces of bone will set all doubt at rest.

Caries or necrosis may often be detected by perforating the antrum and exposing the denuded or diseased bone, or when there is an external opening, by probing it. In this way any loose or dead bone may be felt with the instrument; and the diagnosis in either case will be satisfactory.

When caries or necrosis is situated in the alveolar border, or floor of the antrum, its existence can be more readily ascertained. The occurrence of either in the alveolar ridge, causes the gums to inflame, and to assume a dark purple or livid appearance, to separate from the sockets of the teeth, and frequently to slough off in large pieces and expose the caried or necrosed bone. When situated in the floor of the antrum, the rough denuded bone may be easily felt with a probe or stilet, introduced through the fistula in the gums or alveolus of a tooth from which the matter is discharged.

The pain accompanying these affections does not constitute a diagnosis of much importance, since this is said not to belong to the osseous tissue, but to the soft parts that cover it.

*Causes.*—Caries, necrosis and other alterations of the osseous walls of the maxillary sinus are thought by some, to result, very frequently, from certain specific or constitutional vices; such for example, as the venereal, scorbutic, scrofulous, cancerous, &c. independently of any previous morbid condition of the soft parts. But, I have yet to be convinced, that disease ever occurs in an osseous tissue, except in the teeth, while the soft parts in contact with it, are in a healthy state. I am of the opinion therefore, that the contrary supposition is gratuitous. A bad habit of body or constitutional vice, may perhaps, increase the susceptibility of the bony tissues of the body to morbid impressions, but I do not believe that it ever gives rise, independently of the condition of the soft parts with which they are connected, to actual disease in them.

The immediate cause of caries and necrosis of the osseous walls of the antrum maxillare, is the destruction of their periosteum, caused by inflammation or ulceration; and these last may result from a purulent condition of the secretions of the mucous membrane of this cavity, engorgement, abscess, or from the presence of foreign bodies or tumours, a blow upon the cheek or from other kinds of mechanical violence. They may also result from the irritation produced by diseased teeth, but the pressure of incarcerated fluids may perhaps be regarded as the most frequent cause; and from this too, results some of the most aggravated forms of disease that ever attack the maxillary sinus.

A morbid action kept up in the periosteum for a long time, by ulceration of the lining membrane, or any other aggravated form of disease in the sinus, or neighbouring soft parts, is apt, especially in bad habits, to result in caries of the bone, but when the inflammation is so severe as to cause the immediate destruction of the periosteal tissue, necrosis at once takes place.

The softening of the bone seems to be the result of the action of some solvent fluid upon it, capable of decomposing or breaking down its calcareous molecules. And, although inflammation and ulceration are always present, and appear necessary to the exudation of this fluid, its production nevertheless, seems to be dependent upon some peculiar state or habit of body.

Thus, it is from other affections of this cavity, that those now under consideration are attributable.

*Treatment.*—Complicated, as are most frequently, caries, necrosis and other alterations of the osseous walls of the maxillary sinus, with other affections of this cavity, their cure is often difficult and generally tedious. The first indication to be fulfilled however in their treatment, as in the case of engorgement, and of a muco-purulent condition of the secretions of the sinus, is to obtain free egress for any fluids which may have accumulated in it, and this should be effected in the manner as before described, by the extraction of a molaris or bicuspis, and the perforation of the base of the cavity through its socket. In addition to this, if the disease of the osseous tissue be complicated with any other affection of the sinus, the means necessary for the cure of the disease with which it is complicated, should at once be employed. But it is not necessary here to describe the treatment of the other diseases of this cavity; inasmuch as that has already, or will hereafter be done.

Deschamps, in treating upon the affections of the osseous walls of this cavity, after stating that the perforation or opening into it should be large enough to expose the seat of the disease, recommends the employment of detersive and stimulating injections, a decoction of quinine, tincture of myrrh and aloes, &c. &c. These last, he says, may be introduced into the antrum as injections or by means of pledgets moistened in them. He also directs the cavity to be "cleared of all foreign matter which may have obtained admission into it."* This treatment, having a tendency to promote a healthy action in the lining membrane of the sinus, will often be all that is required; but it should be continued until the caried or necrosed bone has exfoliated, and the secretions of the antrum cease to exhale an offensive odor. The dead bone, however, having exfoliated, a cure is generally soon effected.

But it sometimes happens that the disease of the bone has been produced by some very malignant and incurable affection of the soft parts; in that case, the resources of art, will of course, prove unavailing. And, when the disease of the bone has extended itself to the greater part of the superior maxillary and the bones with which it is connected, as for example, the nasal, palatine, orbital, &c. the most that can be hoped for, from the skill of the physician,

* Maladies des Fosses Nazales, chap. iv. p. 279.

is a palliation of the symptoms. Art, in such cases, can seldom if ever effect a cure, and there are other cases in which it can only retard the progress of the disease, or assist nature in her efforts to separate the dead from the living bone.

It is impossible to lay down rules for the treatment of alterations of the walls of the maxillary sinus, from which it will not be necessary occasionally to deviate. But, it will be sufficient to state, that in those cases where they are extensively involved in caries or necrosis, it will be proper, in addition to perforating the base of the sinus, if by this means the dead bone cannot be so exposed as to enable the surgeon to detach it from the living, to cut away the whole of the alveolar border beneath the cavity, or to penetrate the sinus above it, or even, as Deschamps recommends, "through the cheek itself, whether there be an ulcer penetrating these parts or not." Having by this means exposed the necrosed bone, it should be carefully detached from that which is sound, and removed. By this, the disease interiorly will be more fully exposed, and a better opportunity afforded for applying such other remedies as its peculiar nature may call for. It is important that the sinus should be kept clean, and that the air be kept from it, and whenever any loose pieces of bone are discovered, they should be removed, but their exfoliation should not, as is justly remarked by the author last quoted,* be hastened, by improper interference, unless the state of the patient's health be such as to render it absolutely necessary, for by so doing, a piece of bone that is still attached to the soft parts may be broken. But while this should be carefully avoided, all dead pieces, isolated from the soft parts, should be detached from the sound bone with which it may be connected, and removed.

The character which the affections of this cavity put on, being determined by the state of the constitutional health, or some particular vice of body, it often becomes necessary in their treatment to have recourse to general remedies. If the subject be of a scrofulous or scorbutic habit, or is affected with any specific constitutional vice, such remedies as are indicated by the affection of the general system under which he may be labouring, should be employed. But it is not necessary here to describe the signs by

* Traite des Maladies des Fosses Nazales, chap. iv. p. 281.

which the various habits of body and constitutional vices are designated, nor is it essential to point out the treatment respectively required by each. Full and ample directions upon these subjects will be found in works devoted especially to the affections of the general system.

But although the character and malignancy of the disease are determined by the state of the constitutional health, or disposition of body, its occurrence seems nevertheless, to be dependent upon local irritation. Its continuance also, in many instances, results from this; and the cure, in cases of this kind, soon follows the removal of the cause that gave rise to it. In a case, the history of which I am now about to detail, an example of this sort is furnished.

Case XX. L. S—, a maiden lady of about thirty years of age, of a scorbutic habit, had been affected with pain in her left cheek and alveolar ridge of the upper jaw of the same side, for nearly two years; which at times, had been almost insupportable. Nearly all of her teeth were affected with caries, and from between the necks of several, on the left side in the superior maxillary and gums, a fetid sanies had been exuding for two or three months. Her appetite had become greatly impaired, and a tumour half the size of a black walnut, having formed upon the palatine arch of the affected side, she became alarmed, and in the fall of 1840, came from her residence on the Eastern Shore of Maryland, to Baltimore, in pursuit of medical aid. She appplied to Professor T. E. Bond, who, after investigating her case, and satisfying himself that the affection of the face and mouth was the result of the diseased condition of her teeth, advised her to place herself under my care; which she did, on the following day.

The alveoli of four of the teeth of the affected side, in the superior maxillary, were, on examination, found to be in a necrosed condition, as was also a part of the palatine bone of the same side. The gums around these teeth had separated from the alveolar processes, and had a dark livid appearance. A thin dark coloured, ichorous matter, which when brought in contact with silver, almost instantly turned it black, was constantly exuding from between them and the necks of the teeth. The left nostril was dry, and the opening from the sinus into it had evidently closed, but an exceedingly fetid matter had been discharged from it during the early stages of the disease. The tumour which had formed on the left side of the arch of the palate, was soft and elastic. When pressed, a dark coloured sanies was discharged from the alveoli, and it, for a time, disappeared.

The alveolar processes being in a necrosed and loose condition, it was with some difficulty that I succeeded in removing the bicuspides and the first and second superior molares of the left side, without bringing their sockets away with them. The operation was followed by the discharge of a considerable quantity of fetid sanies; and, in a few days, the alveoli having become completely detached from the sound bone, I removed them, together with a part of the floor of the antrum. The opening thus formed into this cavity was large enough to admit the end of the forefinger. Several small pieces of bone were afterwards exfoliated, from where the teeth had been extracted, and three pieces from the left side of the palatine arch.

Without any other treatment, the place from which the teeth and alveoli had been removed, except the opening that communicated with the maxillary sinus, had in about seven weeks become entirely covered over with firm and healthy granulations. But from the opening into the antrum, a fetid matter was still discharged. This, however, became less and less offensive, until at the expiration of six or eight weeks more, the opening into the nose having become re-established, it lost its fetid odor, and the aperture at the base of the sinus soon after closed.

Thus, in a little more than three months, a complete cure was effected. The patient left the city in the following spring and I have not since heard from her.

The history of the two following cases is taken from Bordenave's Observations on the diseases of the antrum maxillare, as published in the "Memoirs of the Royal Academy of Surgery;"* and although in its translation as here presented, it is considerably abridged, no important fact connected with either, has been omitted.

CASE XXI.—A ribbon-weaver, sometime after having received a blow upon the left cheek below the eye, experienced pain in

* Obs. xix. and xx. vol. 12, 12mo. pages 61 and 64.

his teeth; which was followed by swelling of the cheek and the formation of a tumour beneath the orbit, where he had received the blow. These symptoms yielded to general treatment, but in about a month after, the swelling returned, which was supervened by fever, and a discharge of acrid serous matter in the mouth. These were followed by the formation of a tumour of the palate, from which a large quantity of fetid matter was discharged—an offensive breath, and the removal, by an operation of the economy, of two roots of teeth and one sound tooth. In 1760, Bordenave took charge of the patient, and on examination, found his gums tumefied, black and nearly mortified. The flesh, he says, had separated from the palate bone, and the vitality of the parts was nearly destroyed. The countenance of the patient had a leaden aspect. These symptoms, together with an inspection of the parts, induced M. Bordenave to suspect a disease of the antrum, complicated with a scorbutic vice.

Having satisfied himself with regard to the nature of the affection, he laid bare the decayed bones, and dressed them in a proper manner. Anti-scorbutics were prescribed. Injections were employed, which passed through into the nose. The matter soon became less abundant and of a better quality. The general health of the patient at the same time improved, and the secretions of the antrum were in part discharged through the nose. At the expiration of about six weeks, the exfoliation of nearly the whole of the alveolar portion of the superior maxillary, took place. He could now introduce his fingers into the sinus. Several small pieces of bone were afterwards exfoliated, and by a proper course of treatment, the fleshy portions of the palate, together with the adjoining parts united and closed the opening into this cavity. In a little more than seven months a complete cure was effected.

Case XXII.—A man, whose right superior maxillary at the upper part, had been swollen for about three months, had at the same time, a soft tumour on the interior of the palate, which, on being pressed, caused matter to be discharged from the nostril of that side. These affections, together with tumefaction of the gums, looseness of several of the teeth, and a fetid breath, induced M. Planque, under whose care the patient was placed, to suspect suppuration of the maxillary sinus, complicated with a scorbutic diathesis of the general system. The molares, which only adhered to the gums, having been extracted, matter was discharged through their alveoli. A portion of the maxillary bone was now discovered to be carious, and this, in about a month began to loosen, and a piece about an inch and a half long, and half an inch in width, some time after exfoliated. The tumour exteriorly disappeared; the walls of the sinus approximated, and a cicatrix ultimately closed the opening into this cavity.

The details of many similar cases are on record, but it would be extending the limits of this paper too far, to introduce them here. The history of the cases already given, will suffice to illustrate the treatment of affections of this description. I should however, have given the history of one of mollities ossium of the walls of this cavity, had I not, while treating of ulceration of the lining membrane, quoted a case in which, that affection had become complicated with this.

It sometimes happens that when a very large opening has been formed through the inferior part of this cavity, it does not always readily close. This, however, does not often occur, except the natural opening has become obliterated. But, when the parts do not manifest a disposition to unite, the practice introduced by Bordenave and Scultet,* which consists in cauterizing the interior circumference of the opening, will, in most instances, prove successful. If, however, this and all other means fail, the opening should be closed by means of an obturator of fine gold. This should be accurately fitted to the parts, and secured by means of a broad clasp, to a molar or bicuspid tooth, and if there be none suitable on the side of the mouth to which it is to be applied, the gold should be extended to one on the opposite side. If it be necessary to replace the teeth that have been lost, with artificial ones, these may be so mounted that the plate upon which they are set, shall cover the opening into the maxillary sinus, and thus obviate the necessity of any other obturator.

* Vide, Memoirs de l'Academie Royale de Chirurgie, 12mo. vol. 12, p. 82.

[TO BE CONTINUED.]

